# The confusion effect when attacking simulated three-dimensional starling flocks

**DOI:** 10.1098/rsos.160564

**Published:** 2017-01-18

**Authors:** Benedict G. Hogan, Hanno Hildenbrandt, Nicholas E. Scott-Samuel, Innes C. Cuthill, Charlotte K. Hemelrijk

**Affiliations:** 1 School of Biological Sciences, University of Bristol, Life Sciences Building, Bristol BS8 1TQ, UK; 2 School of Experimental Psychology, University of Bristol, 12a Priory Road, Bristol BS8 1TH, UK; 3 Groningen Institute for Evolutionary Life Sciences, University of Groningen, Groningen, 9747 AG, The Netherlands

**Keywords:** confusion effect, starling flocking, target tracking, realistic three-dimensional computer simulations

## Abstract

The confusion effect describes the phenomenon of decreasing predator attack success with increasing prey group size. However, there is a paucity of research into the influence of this effect in coherent groups, such as flocks of European starlings (*Sturnus vulgaris*). Here, for the first time, we use a computer game style experiment to investigate the confusion effect in three dimensions. To date, computerized studies on the confusion effect have used two-dimensional simulations with simplistic prey movement and dynamics. Our experiment is the first investigation of the effects of flock size and density on the ability of a (human) predator to track and capture a target starling in a realistically simulated three-dimensional flock of starlings. In line with the predictions of the confusion effect, modelled starlings appear to be safer from predation in larger and denser flocks. This finding lends credence to previous suggestions that starling flocks have anti-predator benefits and, more generally, it suggests that active increases in density in animal groups in response to predation may increase the effectiveness of the confusion effect.

## 1. Introduction

Grouping is a common behaviour in many animal taxa and may have a number of benefits for group members. Aggregation can facilitate socialization, mating and locomotive efficiency [1]. Animals may also form groups for predator defence through increased vigilance and dilution effects [2,3]. Furthermore, as prey group size increases some predators suffer lower attack success, a phenomenon that has been termed the confusion effect [4,5]. It has been suggested that this occurs because of increased difficulty in tracking one object among many, a suggestion that has been corroborated in a number of behavioural and computational studies [6–11]. The influence of group density on the confusion effect has received some attention in the literature, but the results are equivocal, with some evidence for an effect [12,13] and some against [7]. Despite this, it has been suggested that the function of observed increases in group density in response to predators may be to maximize the confusion effect [8,14].

Common starlings (*Sturnus vulgaris*) have a tendency to form great aggregations over wintering grounds, and these highly coordinated flocks have been found to have a number of interesting emergent properties [14,15]. Flocks of starlings may represent a good model to understand whether such complicated aggregations benefit from the confusion effect, and whether or not density plays a role in this. The majority of the literature on these starling flocks has concentrated on the mechanisms and phenotype of the behaviour rather than its functions [16–18]. However, Carere *et al*. [19] observed that in areas with high predation pressure, flocks of starlings were larger, denser and escaped predator attacks more often. The authors conclude that this is evidence that flocking serves an anti-predatory function in starlings, which could be brought about by the confusion effect. Further, Zoratto *et al*. [20] observed lower predation success by hawks on flocks than on single starlings, again indicating a function for flocking of predator defence. However, observational studies are somewhat limited in identifying the specific mechanism or mechanisms through which increased group size or density could lead to reduced predation success.

Empirically investigating the incidence of the confusion effect in animals in the wild is difficult due to logistics, ethical concerns and other constraints, particularly when animal groups are large or mobile. This has led researchers to methodologies using computerized stimuli with human participants as ‘predators’. These experiments closely control the stimuli presented to the participant, such as the size, density and coloration of the group of prey. However, to date, most computerized studies of the confusion effect have used small groups of prey in two-dimensional environments with movement that is relatively simple. This has facilitated our understanding of the underlying rules of the confusion effect, for instance the effects of prey coloration [21–23], and the differential effects of density and number [12,13]. However, more realistic simulations are necessary in order to increase our understanding of which specific properties of aggregations of animals may influence the likelihood and degree of visual confusion in predators. For instance, as a predator approaches realistic prey groups, the apparent size, and the number of individuals in the predator's field of view will change, and it is not known how or if this will influence the confusion effect.

StarDisplay is a realistic computational model of three-dimensional, collective motion of starlings in flocks, and is therefore suitable for such an experiment. This model uniquely reproduces empirical details of the three-dimensional shape, density and internal structure of the flock [17,18,24]. In order to study the influence of the confusion effect on a (human) predator when attacking flocks, we have adapted the model by making it possible for a participant to control a predator while attacking a simulated flock. In this setting, we investigated the influences of the size and density of the flock on the ability of a ‘predator’ to visually track and capture a target by clicking a trigger when close to it. The results will inform us about the likelihood that starling flocks confuse visual predators and whether flock density influences this confusion. We predict that increasing flock size should increase difficulty in tracking, thereby increasing targeting errors. However, we do not have specific hypotheses for flock density, as there is conflicting evidence for the influence of density on the confusion effect, and any interaction with group size in this respect.

## 2. Results

**Figure 1.**
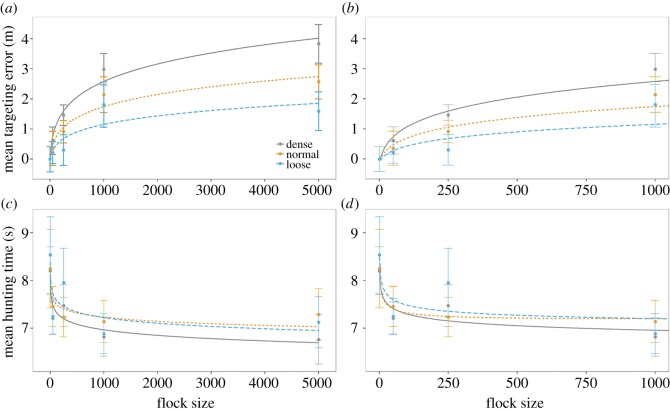
(*a,b*) Graphs of participant mean targeting error plotted against the natural logarithm of flock size. (*c,d*) Graphs of participant mean hunting time plotted against the natural logarithm of flock size. The leftmost images display the whole dataset; the rightmost are zoomed into flock sizes 1–1000. Line colour and solidity indicate flock density, and error bars indicate within-subject 95% confidence intervals, fitted lines are quadratic polynomials.

For targeting error, the most complex statistical model was one where both flock size and flock density are fitted as quadratic polynomials; we tested this model against simpler models where these effects were modelled as linear terms. A model where flock size was fitted as a linear term explained significantly less deviance than one with a quadratic polynomial fit of flock size (*χ*^2^ = 28.6, d.f. = 3, *p* < 0.001). A model where flock density was fitted as a linear term was not significantly worse than one where flock density was fitted as a quadratic polynomial (*χ*^2^ = 0.61, d.f. = 3, *p* = 0.09), but because it has equivalent predictive power and fewer terms the linear model is the preferred model. Therefore, in all subsequent models, flock size was fitted as a quadratic polynomial, and flock density was fitted as a linear term. There was a significant interaction between flock density and flock size for targeting errors (*χ*^2^ = 21.26, d.f. = 2, *p* < 0.001). This indicates that the effect of flock size changes with flock density, and that the effect is parsimoniously described as linear. Targeting error increases with both flock size and flock density, and at higher densities the effect of flock size increases (figure 1). Further, the effect of flock size on targeting error was tested for each level of density with independent models: there was a significant effect of flock size for dense (*χ*^2^ = 22.2, d.f. = 2, *p* < 0.001), normal (*χ*^2^ = 29.9, d.f. = 2, *p* = 0.007) and loose conditions (*χ*^2^ = 12.7, d.f. = 2, *p* = 0.002).

A model fitting hunting time against flock density and a quadratic polynomial of flock size did not explain a significantly different amount of deviance than one with only linear terms (*χ*^2^ = 3.18, d.f. = 3, *p* = 0.364), so subsequent models fitted flock size as a linear term. For hunting time, the flock size × flock density interaction was not significant (*χ*^2^ = 0.81, d.f. = 2, *p* = 0.668). The main effect of flock density was not significant (*χ*^2^ = 2.54, d.f. = 2, *p* = 0.281), but the main effect of flock size was significant (*χ*^2^ = 41.77, d.f. = 1, *p* < 0.001; figure 1). Thus, the average hunting time was shorter for larger flocks.

**Figure 2.**
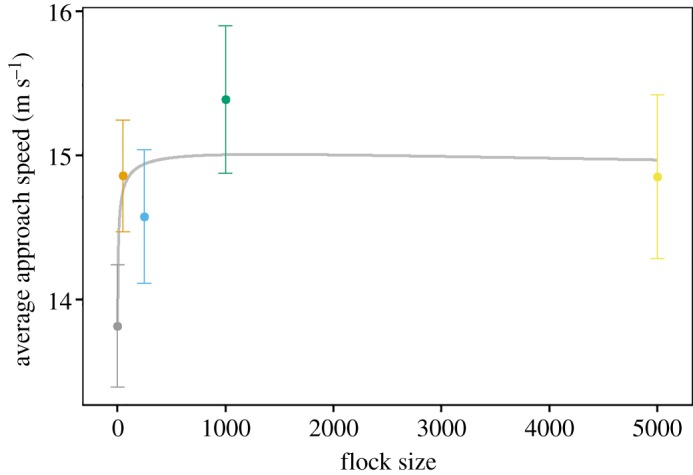
Graph of participant mean approach speed plotted against the natural logarithm of flock size, the fitted line is a quadratic polynomial, error bars indicate within subject 95% confidence intervals.

We next investigated whether participants may be taking different strategies to attack targets dependent on flock size and density. In order to investigate whether and how participant movement speed and approach speed (the average speed of the reduction in distance between the predator and prey over the trial) depended on flock density and size, their average values were analysed. A model where average predator movement speed was fitted against flock density and a quadratic polynomial of flock size was not significantly better than a similar model where flock size was fitted linearly (*χ*^2^ = 5.1, d.f. = 3, *p* = 0.16), so subsequent analyses fitted number linearly. The flock size × flock density interaction for movement speed was not significant (*χ*^2^ = 0.42, d.f. = 2, *p* = 0.81), nor was the main effect of flock density (*χ*^2^ = 4.59, d.f. = 2, *p* = 0.1), or the main effect of flock size (*χ*^2^ = 0.75, d.f. = 1, *p* = 0.39). This indicates that participant average movement speed was not significantly influenced by flock size or density. Turning to approach speed; a model fitting approach speed against flock density and a quadratic polynomial of flock size was significantly better than a similar model with a linear fit of flock size (*χ*^2^ = 14.79, d.f. = 3, *p* = 0.002), so subsequent models fitted flock size as a quadratic polynomial. The flock density × flock size interaction for approach speed was not significant (*χ*^2^ = 2.52, d.f. = 4, *p* = 0.64). The main effect of flock density was not significant (*χ*^2^ = 1.19, d.f. = 2, *p* = 0.56), but the main effect of flock size was significant (*χ*^2^ = 16.64, d.f. = 2, *p* < 0.001; figure 2). Thus, targets in larger flocks were approached at higher speeds (although see the electronic supplementary material).

Our results therefore indicate that both flock size and density increased participants' tracking error. Further, flock size influenced participants' hunting time: participants were faster to click when target prey were in larger flocks. Examination of the participants' average movement speed and approach speed indicates that this reduced hunting time may have been caused by faster approach speeds when the target prey were in larger groups. Although there was no implementation of different motion for prey depending on group size, further analysis indicates that prey motion did vary between groups (see the electronic supplementary material). We investigated whether this may affect the confusion of the predator. Several aspects of prey motion that may influence capture were measured, namely the average and variation of the prey speed, prey acceleration and the curvature of prey trajectory. The results show that prey in denser groups took more curved paths, and had greater linear acceleration, than targets in less dense groups (see electronic supplementary material, table S2). Prey curvature and linear acceleration peaked at groups in the range of 50–250, and was lower for solitary prey and larger groups (see electronic supplementary material, figure S4). Targets in larger groups moved slightly more slowly than those in smaller groups (see electronic supplementary material, figure S4). These differences could have contributed to the confusion of predators. Our analysis (see electronic supplementary material, table S1) suggests that these differences in prey behaviour do not explain participant tracking error or hunting time better than flock size and density *per se*. However, we cannot rule out these factors as an additional cause of confusion for participants. Further, we found that participant approach speed was better explained by the curvature and variation in the curvature of the prey trajectory, and by prey speed and prey variation in linear acceleration, than by flock size (see electronic supplementary material, table S1). This indicates that the speed at which predators approached prey is better explained by these parameters of the prey's movement than by the size of the flock. The findings indicated that increased curvature, variation in curvature and variation in linear acceleration increased predator approach speed, and increased prey speed decreased predator approach speed.

## 3. Discussion

The results are consistent with suggestions that large aggregations of starlings have anti-predatory functions through the confusion effect. As flock size increased, targeting errors increased. In addition, this effect significantly interacted with flock density, leading to greater effects of flock size on targeting error in denser groups (and *vice versa*). The pattern of results for hunting time is intriguing; participants took significantly longer to attack birds in smaller groups. Analysis of movement patterns of predator and prey indicates that this may have been due to more direct approaches to targets in larger flocks, or by differences in prey movement between flock sizes, or both, rather than faster predator movement speed.

In line with predictions from the confusion effect, participant error in attempting to capture a starling in a flock increased with flock size. This lends evidence to suggestions that the tendency to form large aggregations over wintering grounds in starlings has an anti-predatory function [19,20,25]. Our experiment has shown that as flock size increases, predators may find it more difficult to catch a given individual. This result is consistent with observational studies on starlings which have found that predator attack success is lower against larger flocks and larger flocks are more common where predation pressure is high [19,20]. In a more general sense, this finding also corresponds with behavioural experiments on the confusion effect in other predator–prey systems [4,7,9,11]. It may therefore be reasonable to predict that reduced attack success against a predetermined individual extends to reduced success without this constraint and indeed where predators switch targets, or are forced to through confusion, they may pay switching costs [26], for instance by rapidly changing direction or speed. Interestingly, the increase of error with flock size does not appear to have reached a plateau at 5000 birds, which may indicate that starlings could benefit by forming aggregations even larger than this. However, there do appear to be diminishing returns for flock size, with greater benefit accrued by increases in group size for small groups, and the costs of forming aggregations (e.g. increased predator attack rate and competition [1]) are also likely to constrain real group sizes. Further research on the influence on confusion of flock sizes between 1000 and 5000 birds could benefit our understanding of the benefits of such large and complex aggregations.

Evidence for the influence of density on the confusion effect from computerized experiments has been conflicting, with some evidence for a role [12,13], and some against [10]. Our experiment demonstrates that in a dynamically simulated scenario there is a significant influence of flock density on the participant's tracking errors. Errors were higher for denser prey flocks, and this effect interacts with flock size (and therefore the confusion effect). Again, this corresponds well with observational studies on starling flocks, where denser and larger flocks are found where predator pressure is high [19]. This may also bear upon understanding of the reactions of prey in groups under predation. Starlings [19], and a number of shoaling fish species [27,28], are known to increase group density in response to the presence of a predator. Additionally, it has been found that starling flocks are more densely packed at the periphery than the centre of the flock [14]. It has been suggested that these may constitute behavioural responses that act to maximize the deleterious influence of the confusion effect [8,14]. Our results are consistent with this idea, and overall the results suggest that larger and denser flocks of starlings cause more visual confusion in predators.

In contrast with Ruxton *et al*. [10], who found that participants took longer to catch individuals in larger groups, participants in our experiment were significantly quicker to attack targets in larger groups. This may be related to the fact that Ruxton *et al*. used a binary coding of hits and misses, rather than a continuous measure of error as used here, and reported time to capture only for those trials where a target was considered caught. Therefore in the experiment by Ruxton *et al.* [10], increased time taken to capture individuals with increasing prey group size may indicate increasing difficulty in tracking prey in larger groups. In our experiment, hunting time was analysed for all trials regardless of error, so could reflect a trade-off between accuracy and speed in the participant's approach to predation. Lower hunting time for larger groups may relate to reduced motivation of participants to approach slowly and carefully in trials with large flock size, due to an increase in the chance of losing track of the target. In these trials, participants may have taken a strategy of more direct and less precise attacks rather than risk more measured strategies because the probability of losing the prey accumulates over time. The results of the analysis for participant average speed and approach speed may support this suggestion. Participant movement speed was not influenced by flock size, but approach speeds were significantly higher when attacking flocks than when attacking singletons. This may indicate that participants took more direct approaches when targets were in groups. However, further analysis of the behaviour of prey in flocks (see the electronic supplementary material) indicates that the differences in participant approach speed may have instead been driven by differences in the movement of prey across flock size and density. We found that prey with more curved and more variably curved trajectories and those with greater variation in linear acceleration were approached at higher speeds, and prey moving at higher speeds were approached more slowly.

The methodology chosen in the current study was deliberately geared towards investigating the confusion effect in a controlled way. For instance, we initialized the locations of the predator and prey at the same altitude and distance apart from each other in each trial in order to allow comparisons of hunting time. We also predetermined the identity of the target in order to understand how the ability of the predator to track an individual varies with flock size and density. However, this predetermination also meant that in many cases the predator was obliged to move through the flock to capture the target, whereas real predators tend to attack from the periphery [20]. Future research should alter or remove these constraints, in order to simulate more realistic predator behaviour. Allowing participants to target all model starlings freely would allow the investigation of the overall features of the attack strategies chosen by participants, and reveal whether their trajectories differ when attacking large or small groups, as well as how this relates to data on the attack strategies of real predators. In this study, simulated starlings did not respond to predator attack, but the behaviour of individual starlings did vary across flock sizes and densities (because they respond to one another). While further analysis (see the electronic supplemental material) indicates that emergent differences in individual prey behaviour did not explain the confusion effects better than flock size or density, we cannot rule out the possibility that individual behaviour may contribute to the confusion effect. Previous empirical work on the confusion effect in computerized experiments indicates that individual differences in behaviour in groups are not necessary for the confusion effect to occur [10,13,22], but in reality individuals in groups may often move somewhat differently and these differences may contribute to the confusion of the predator: this would be an interesting avenue for future research.

In summary, previous computerized experiments on the confusion effect have largely used simple two-dimensional environments with relatively simplistic schemes of movement. The current experiment aimed to investigate the influence of flock size and density on the ability of participants to accurately track and capture targets in dynamically and realistically simulated three-dimensional starling flocks. In line with observational evidence in starlings, as well as behavioural and computerized studies on other predator–prey systems, predator attacks were less successful against larger groups of prey. Additionally, increasing density of the prey group impeded the predators' ability to track and capture targets, an effect which was enhanced with increasing group size. The confusion effect in visual predators makes starlings safer when they are in large, dense flocks.

## 4. Material and methods

There were 25 participants. They were recruited through a research participation scheme in the School of Experimental Psychology at the University of Bristol. The median age of the participants was 20 years, and there were 11 females and 14 males.

The StarDisplay model was used to represent starling flocks. Its behavioural rules are animal-centred in that individuals are made to coordinate with their seven closest neighbours in line with empirical observations [29], and individuals follow simplified flying behaviour parametrized to starling flight. Flying behaviour has been shown to be essential for generating the variation of flock shapes as observed empirically [17]. The modelled flocks resemble real flocks in many traits: shape and orientation of the flock; aspects of turning, such as maintenance of shape during a turn; the change of the orientation of the shape relative to the movement direction and the repositioning of individuals during turns [17]; the degree of diffusion in the flock [18]; the scale-free correlation between the absolute length of the flock (in metres) and the correlation length of the deviation of the velocity and of speed of individuals from the velocity and speed of the centre of gravity [24].

**Figure 3.**
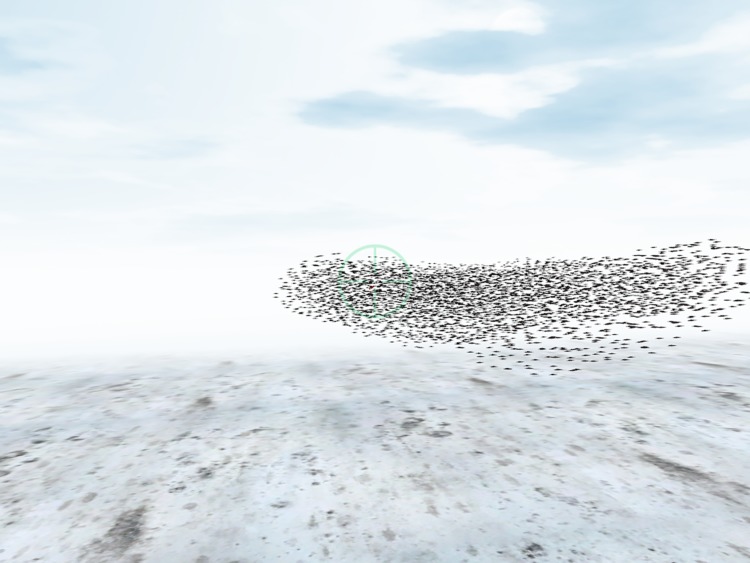
Screenshot representative of participant view during experimental trials.

The StarDisplay model was used to create a ‘game’ style experiment separated into trials, where in each trial a participant attacks a target in a flock of specific size and density. The experimental setting resembles that of a flight simulator used in games. The human steers an avian predator by means of a game controller while observing the three-dimensional environment via a virtual camera from the perspective of the virtual bird (figure 3). The mechanics of the flight of the predator were simplified compared with those of the model starlings, in order for the participants to control the predator easily. Simulated starlings did not react to the predator in any way.

At the onset of each trial, participants were presented with a starling flock, with an on-screen crosshair pointing toward a randomly selected target in the flock. The Euclidian distance from the position of the predator to the target prey at the onset of each trial was fixed at 100 m, and the predator and prey began at an identical altitude of 50 m within the simulation. Upon clicking the trigger button of the joystick, the trial began, and the simulation started to run in real time. At this point, the participant could steer the predator and control its speed using the joystick by adjusting roll, pitch and thrust of the predator. Participants were tasked with approaching the target, and clicking again when they felt that they were as close as possible to ‘catching’ the target, which ended the trial. During the trial, the target starling was identified by a red trail, which joined all locations the starling had been for the interval of one second. When the distance from the predator to the target prey reached 40 m, this trail became linearly more transparent until a distance of 20 m, after which it was invisible. If the participant did not click within 30 s of the start of the trial, the trial timed out, and was excluded from analysis. The participant then received feedback on their distance to the target starling during the trial, along with a qualitative statement (‘Direct hit!’ within 0.005 m, ‘That was a catch!’ 0.005–0.02 m, ‘That was nearly a catch!’ 0.02–1 m, ‘Somewhat close to the target’ 1–2 m, ‘You missed your target’ 2–10 m). After this, the participants could click to begin the next trial.

The experiment included a fully factorial combination of flock size (1, 50, 250, 1000 and 5000) and flock density (‘loose’, ‘normal’ and ‘dense’, representing 0.8 m, 1.3 m and 1.8 m nearest neighbour distance on average). The density range covered that reported for real starlings [14,30]. Each participant completed a practice period in which all combinations occurred four times, with the order of trials randomized independently for each participant. In the experimental period, each combination occurred 15 times and the order of occurrence of all trials was completely randomized, independently for each participant. The practice period took approximately 20 min to complete, and the experimental period took approximately 1 h to complete. Participants were free to take short breaks between trials. All stimuli were viewed at 62 cm from a gamma-corrected 19^″^ Dell Trinitron CRT monitor (Dell Inc., Round Rock, TX, USA), with refresh rate 100 Hz, a resolution of 1024 × 768 pixels and mean luminance of 71.4 cd m^−2^. At the experimental viewing distance, each pixel subtended 2.2 minarc. The joystick used to control the predator was a Logitech Extreme™ 3D Pro (Logitech International S.A., Lausanne, Switzerland).

All statistical analyses were performed in R (R Foundation for Statistical Computing, www.R-project.org). The analysis used was a combination of traditional hypothesis testing and model fitting using general linear mixed models (function lmer in the lme4 package; [31]). Relaxing the compound symmetry assumption for this repeated measures design, by use of generalized least-squares (function gls in package nlme; [32]), produced a very similar result in terms of effect sizes and statistical significance, so we present the simpler analyses here. The most complex model fitted flock size as a quadratic polynomial along with flock density and includes all factors and their interactions; subsequent statistical models address whether main or interaction effects can instead be modelled as linear terms. Subject was a random effect in all models. The change in deviance between models with and without the predictor variables of interest was tested against a *χ*^2^ distribution with degrees of freedom equal to the difference in degrees of freedom between the models [33].

The Euclidean distance from the target at which a participant clicked the trigger (targeting error) was used as a continuous rather than a binary outcome for our experiment because this more accurately encompasses the variation in participant tracking errors. A binary response may have eliminated any differences in error caused by the timing or degree of tracking mistakes. Continuous errors mean that the data show differences between complete failures and near misses, and thus present richer information. Data from the practice period were not analysed. All trials where targeting errors were greater than 20 m from the target were discounted as mechanical, hardware or human errors and therefore not analysed (*n* = 267 trials, 5.9% of overall). Twenty metres was chosen because visual inspection indicated that targeting errors depart from a normal distribution at this point, and because it is more than three times the average clicking error in the conditions with the highest error. Targeting errors were normalized by subtraction of the average error during conditions where there was only a single starling (for each participant individually); this eliminated individual differences in the component of error caused by the mechanically difficult nature of the experiment, and means that all non-zero error was a result of increased group size. The time taken from the initialization of a trial until a participant clicked (hunting time) was also recorded and analysed, as was the participant's average movement speed within each trial. Participant average approach speed was also recorded: this was calculated by finding the mean of the reduction in distance between the predator and target in each frame across the whole trial. The distribution of targeting errors, hunting time and approach speed were approximately log normal with respect to flock size, so all analyses on these measures use a natural logarithmic transformation of flock size.

## Supplementary Material

PDF document containing: further analysis, table 1, table 2, figure 4.

## Supplementary Material

Data File; Text file containing all participant data

## Data accessibility

The datasets supporting this article have been uploaded as part of the electronic supplementary material.
