# Supplementary material for: The confusion effect when attacking simulated three-dimensional starling flocks
Source: R Soc Open Sci. 2017 Jan 18;4(1):160564. doi: 10.1098/rsos.160564 (PMC5319319; doi:10.1098/rsos.160564)
Supplement: PDF document containing: further analysis, table 1, table 2, figure 4. [file rsos160564supp1.pdf]

Further analysis of prey movement within flocks of varying size and density was undertaken. Fifty flocks were simulated for each combination of flock size and density, and the prey behaviour over the first 7.5 seconds (the average trial length during the experiment) was recorded and averaged. We calculated the curvature of the prey trajectory to understand if prey in larger flocks took more complex paths. Curvature was calculated with the following formula, where r is the position at time t, r' the velocity, r'' the acceleration:

$$\mathbf{K}(\boldsymbol{t}) = \frac{\left|\overrightarrow{\boldsymbol{r}'}(t) \times \overrightarrow{\boldsymbol{r}''}(t)\right|}{\left|\overrightarrow{\boldsymbol{r}'}(t)\right|^{3}}$$

In addition, in order to study whether the motion of prey differs between group sizes and densities, the mean and standard deviation of prey speed and linear acceleration was measured. We found that prey movement did vary by flock size and density (see Table 2).

To investigate whether these measures of prey movement, rather than group size or density *per se*, better predicted the patterns of targeting error and hunting time further analysis was undertaken. Comparisons of the AIC values of independent Generalised linear models each using one prey movement measures to predict targeting error and hunting time, against those selected in the main analysis indicate that no prey movement measures predict targeting error or hunting time better than flock size or density (see table 1). This indicates that prey behaviour did not have greater influence on hunting time or targeting error than group size or density. However, the speed at which participant approached targets was better predicted by some prey movement measures than by group size. The average approach speed of the predator appears to have been influenced positively by increases in curvature and the variation in curvature of prey trajectory, as well as by variation in linear acceleration, and negatively by prey speed.

Table 1 – AIC values for generalised linear models fitting targeting error, hunting time, and participant average approach speed, against each prey behavioural measure individually (with participant as a random factor). These values can be compared to the AIC value of the selected models from the original analysis. Values of AIC that are lower than those from selected models suggest that the prey behavioural measure had more influence than the factors in the selected statistical model. For targeting error, the selected model fitted error against flock density and a quadratic polynomial of flock size, with the two way interaction between these factors, and had AIC=1303.723. For hunting time, the selected model fitted hunting time against a quadratic polynomial of flock size, and had AIC=1353.875. For approach speed, the selected model fitted approach speed against a quadratic polynomial of flock size, and had AIC=-65.588. Values of AIC which are lower than those of the respective selected model from the original analysis are in bold.

		Targeting Error	Hunting Time	Approach Speed
Prey Speed	Mean	1366.303	1370.946	**-65.898**
	SD	1508.664	1393.793	-56.597
Curvature	Mean	1495.133	1380.974	**-69.747**
	SD	1499.301	1374.663	**-77.117**
Linear Acceleration	Mean	1495.577	1384.699	-57.387
	SD	1456.077	1369.446	**-66.831**

Table 2 - Table of measures of prey movement by flock size and density; values represent the mean and standard deviation from 50 simulated flocks of each combination of flock density and size.

Flock		Prey Speed (m/s)		Curvature (1/m)		Linear Acceleration (m/s^2)	
Size	Density	Mean	SD	Mean	SD	Mean	SD
1	dense	13.6194	0.0177	$1.93*10^{-5}$	$1.76*10^{-6}$	0.0934	0.0033
1	normal	13.6201	0.0346	$1.98*10^{-5}$	$4.33*10^{-6}$	0.0964	0.0106
1	loose	13.6062	0.0081	$1.86*10^{-5}$	$9.64*10^{-7}$	0.0936	0.0064
50	dense	13.6123	0.0564	$4.36*10^{-5}$	$3.38*10^{-6}$	0.9382	0.1647
50	normal	13.5990	0.0417	$3.33*10^{-5}$	$3.27*10^{-6}$	0.5275	0.1095
50	loose	13.6028	0.0322	$2.68*10^{-5}$	$3.13*10^{-6}$	0.3094	0.0557
250	dense	13.6049	0.0321	$3.74*10^{-5}$	$3.44*10^{-6}$	0.7786	0.1301
250	normal	13.6143	0.0213	$2.91*10^{-5}$	$2.59*10^{-6}$	0.3928	0.0645
250	loose	13.6039	0.0235	$2.46*10^{-5}$	$3.86*10^{-6}$	0.2634	0.0487
1000	dense	13.5133	0.0223	$2.43*10^{-5}$	$3.67*10^{-6}$	0.6982	0.1283
1000	normal	13.5108	0.0230	$1.42*10^{-5}$	$3.30*10^{-6}$	0.3232	0.0863
1000	loose	13.5150	0.0210	$9.66*10^{-6}$	$2.08*10^{-6}$	0.1942	0.0567
5000	dense	13.5242	0.0211	$2.37*10^{-5}$	$3.14*10^{-6}$	0.6772	0.1551
5000	normal	13.5168	0.0194	$1.50*10^{-5}$	$2.30*10^{-6}$	0.3261	0.0760
5000	loose	13.5273	0.0183	$9.71*10^{-6}$	$1.66*10^{-6}$	0.1882	0.0550

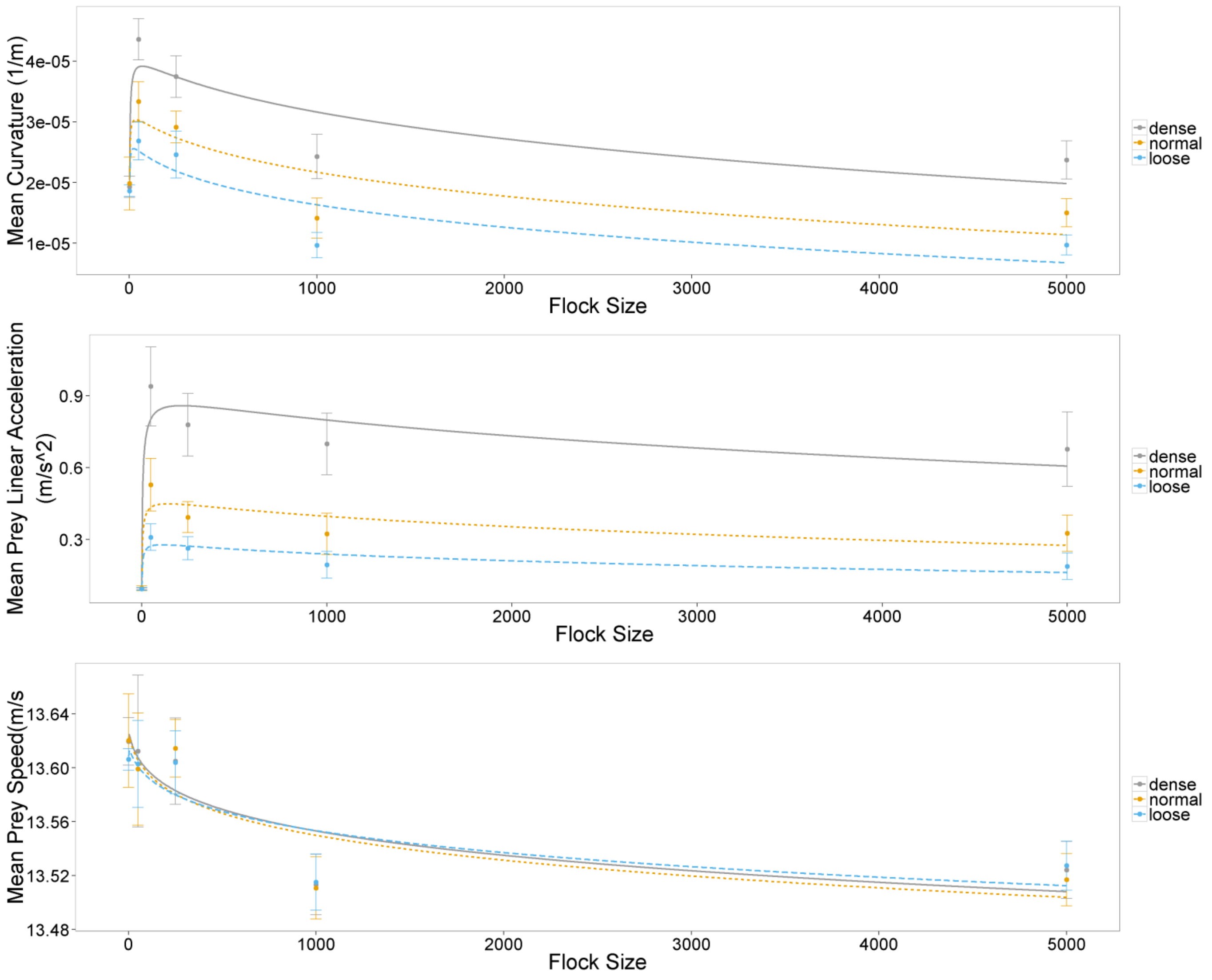

Figure 4- Graphs of prey bird movement measures by flock size and density, fitted lines are quadratic polynomials. Line colour and solidity indicate flock density. Error bars indicate standard deviation.
